# Fluid–structure interaction analysis of aortic valve dynamics: effects of aspect ratio

**DOI:** 10.1093/rb/rbag167

**Published:** 2026-08-05

**Authors:** Xiyue Zhang, Liangqi Zhang, Zhong Zeng, Yibo Han, Xing Zhang, Xueli Wang, Quanchao Zhang, Guixue Wang

**Affiliations:** Department of Engineering Mechanics, College of Aerospace Engineering, Chongqing University, Chongqing 400044, China; Department of Engineering Mechanics, College of Aerospace Engineering, Chongqing University, Chongqing 400044, China; Department of Engineering Mechanics, College of Aerospace Engineering, Chongqing University, Chongqing 400044, China; Key Laboratory for Biorheological Science and Technology of the Ministry of Education, National Local Joint Engineering Lab for Vascular Implants, College of Bioengineering, Chongqing University, Chongqing 400044, China; Institute of Metal Research, Chinese Academy of Sciences, Shenyang 110016, China; Wuhan Vickor Medical Technology Co. Ltd., Wuhan 430205, China; Nanchang Key Laboratory for Smart Biomaterials Regulation and Adaptation & School of Materials Science and Engineering, East China Jiaotong University, Nanchang 330013, China; Key Laboratory for Biorheological Science and Technology of the Ministry of Education, National Local Joint Engineering Lab for Vascular Implants, College of Bioengineering, Chongqing University, Chongqing 400044, China

**Keywords:** prosthetic aortic valve, fluid–structure interaction, arbitrary Lagrangian–Eulerian, aspect ratio, hemodynamics

## Abstract

The geometric configuration of prosthetic aortic valve is a key determinant of its mechanical response and associated hemodynamics during the cardiac cycle. In this work, high-fidelity fluid–structure interaction (FSI) analysis is conducted to provide physical insight into the effects of geometry on valve design. We integrate the data from the Doppler experiments and the Windkessel model for the inlet velocity and outlet pressure conditions, respectively. The arbitrary Lagrangian–Eulerian method is employed to address the two-way interaction between hyperelastic leaflets undergoing periodic large deformation and the surrounding blood flow. The effects of leaflet aspect ratio (AR) and blood non-Newtonian properties on mechanical performance are systematically investigated. The FSI analysis shows that increasing AR elevates von Mises stress, wall shear stress, blood velocity magnitude, transvalvular pressure gradient and regurgitation fraction (RF), while reducing geometric orifice area (GOA). These trends are opposite to the results from pure structural mechanical analysis. As AR increases from 0.86 to 1.26, the maximum von Mises stress increases by 58.66%, whereas GOA decreases by 71.54%. For the AR = 1.26 valve, the jet deviates from the centerline toward the lower vessel wall during peak systole and the subsequent deceleration phase, resulting in asymmetric deformation.

## Graphical abstract

**Figure:**
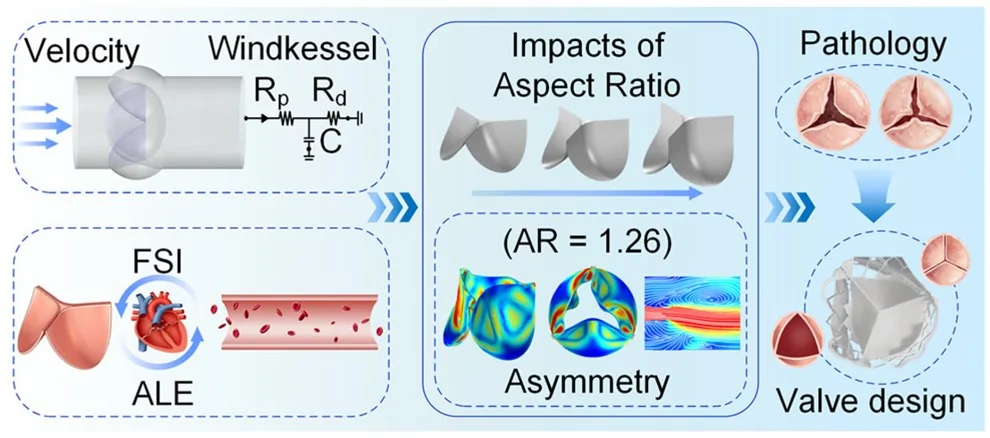

## Introduction

Aortic valve (AV) disease has become the second leading cause of mortality among cardiovascular diseases because of its high incidence and associated death rates [1], posing a serious threat to human health. The major pathological features of AV disease include valve calcification, stenosis, regurgitation, etc. [2]. Valve replacement surgery has been proven to be an effective clinical treatment for severe heart valve disease [3]. Currently, mechanical and bioprosthetic valves are two primary solutions. Mechanical valves have the advantages of excellent durability and longevity, but patients require lifelong anticoagulant medication after surgery and face a risk of thrombosis [4]. Biological valves offer superior biocompatibility and hemodynamic performance, significantly reducing anticoagulant-related complications [5]. Nevertheless, they are prone to calcification or degradation of fibrous structures during prolonged in-body service, limiting their operational lifespan. Therefore, despite the substantial progress reached, existing prosthetic heart valves still face notable challenges in long-term durability, calcification resistance, hemocompatibility and the avoidance of anticoagulation therapy [6]. The interest in exploring artificial valve materials and optimizing the geometric design is increasing, which can benefit from an in-depth understanding of the interaction between highly deformed valve leaflets and hemodynamics.

Attempts have been made to analyze the fluid–structure interaction (FSI) of the valve through numerical simulations. Key parameters, such as the geometric orifice area (GOA), regurgitant fraction (RF), transvalvular pressure gradient (TPG) and wall shear stress (WSS), are obtained from the computational results to reflect the mechanical properties and hemodynamic state [7–12]. Specifically, the GOA and TPG jointly evaluate valve flow efficiency to determine the risk of AV stenosis [13]; abnormalities in valve leaflet stress and average WSS (AWSS) are closely correlated with endothelial injury and calcification [14, 15], and the RF reflects whether the valve closes completely [16, 17].

The interaction between valve deformation and blood flow over a complete cardiac cycle involves complex nonlinear coupling. This complexity poses significant challenges to accurate mathematical modeling and numerical simulation. Early studies often relied on pure structural mechanical analysis to avoid such complexity. Examples include the work by Weltert et al. [18] and Capelli et al. [19]. They simplified the leaflets as linear elastic shells to predict the stress distribution. At present, a range of numerical approaches have been developed to address the challenges of FSI between the valve and blood flow. Among them, the smoothed particle hydrodynamics (SPH) method [20–22], the immersed boundary (IB) method [23–26] and the arbitrary Lagrangian–Eulerian (ALE) method [27–29] are the most widely used methods. The SPH method provides a unified Lagrangian description for both fluid and solid regions, and particle-based mesh-free discretization avoids complicated grid updating and processing. However, robustness, accuracy and conservation issues may arise in SPH-based FSI analysis because of improper boundary condition treatments and tension instability. The latter two approaches both rely on the mature finite element method solver for the stress and deformation prediction of the solid region, thus providing quantitatively reliable results.

By using the IB method, Black et al. [30] investigated the FSI between the hyperelastic valve and blood in a two-dimensional configuration. For three-dimensional attempts, the valves were typically modeled as hyperelastic solid elements. Lopez et al. [31] conducted FSI analysis by combining a time-varying inlet velocity and a constant-pressure outlet boundary condition. Nestola et al. [23] also used a time-varying velocity as the inlet boundary condition while modifying the outlet boundary treatment by referring to the Windkessel model, which better fits the physiological pressure response. Cai et al. [32] employed the Windkessel model to describe the outlet pressure boundary condition and used a pressure profile as the inlet condition to analyze AV dynamics. As a direct extension of the work by Cai et al. [32], Halevi et al. [33] used shell elements to model the valve and simulated the dynamic opening and closing process of the leaflets over 0.32 s (two-fifths of a complete cardiac cycle). Notably, the IB method resolves FSI by facilitating data communication between a Lagrangian grid representing the solid component and a stationary background Eulerian grid for the fluid domain, typically through specialized force distribution and velocity interpolation kernels. However, when the solid structure experiences large deformation, the nonconformal nature of these grids often leads to numerical dissipation and a failure to strictly satisfy mass and momentum conservation at the interface, which consequently degrades the solution accuracy.

The ALE method provides a more rigorous framework by explicitly aligning the mesh with the phase boundaries. It directly imposes FSI conditions at the fluid–structure interface using a moving mesh technique to track the interface, thus proving that the ALE method is the most accurate approach for FSI problems involving significant structural deformations. Kivi et al. [34] used a linear elastic leaflet model to investigate hemodynamics in a two-dimensional configuration. In the two-dimensional analysis by Anwar et al. [35], the valve leaflets were hyperelastic and deformed under a steady-state assumption. For three-dimensional configurations, Zhu et al. [36] used linear elastic solid elements to describe leaflet deformation and applied a velocity inlet boundary condition. They only analyzed the dynamic deformation of the leaflets over 0.08 s. Liu et al. [37] modeled the valve as hyperelastic shell elements and performed FSI simulations over a cardiac cycle using pressure difference conditions between the inlet and outlet to analyze the hemodynamic performance. Li et al. [38] defined the valve as hyperelastic solid elements, applying a transient Doppler-measured velocity profile at the inlet and setting the outlet to zero pressure. Nowak et al. [39] adopted a pulsatile pressure condition at the outlet, but they were unable to complete a full cardiac cycle simulation because of prohibitively excessive computational costs. In contrast, Hart et al. [40] simplified the leaflets as linear elastic solid elements, enabling an analysis of leaflet deformation and blood flow evolution over an entire cardiac cycle.

With the extensive development of numerical methods such as IB and ALE, geometric design of the AV has also become an increasingly important research focus. Leaflet height and radius are two key geometric parameters that determine the long-term functional stability of prosthetic AVs. An appropriate leaflet height is essential for maintaining sufficient leaflet coaptation. In contrast, an inappropriate leaflet height can disrupt the geometric balance of the valve, impair leaflet closure and increase the risk of regurgitation, highlighting the importance of leaflet height adjustment in prosthetic valve design and clinical assessment. Since the leaflet radius of the native AV is relatively consistent, variations in leaflet height represent a critical geometric factor influencing valve function [41–44]. However, few studies have systematically investigated the effects of the leaflet height and radius in existing numerical simulations of valve–blood interactions. Hou et al. [43] investigated hyperelastic leaflet behavior using a shell model, focusing on the effects of leaflet height and radius. Their results showed that, at a fixed radius, increasing height improves mechanical performance. Nevertheless, fluid effects were not considered, limiting the ability to capture physiological motion under realistic hemodynamic conditions. Ma et al. [44] performed an FSI analysis using a hyperelastic solid model. They reported that increasing height leads to deteriorated hemodynamic performance. Despite incorporating FSI, the use of solid elements representation neglects the thin-shell nature of leaflets. In addition, the inlet and outlet boundary conditions adopted in their study were highly simplified and did not adequately represent the physiological loading conditions experienced by the AV. Overall, existing studies either ignore FSI or do not properly account for the thin-shell characteristics of leaflets, or rely on simplified physiological boundary conditions, leading to inconsistencies in describing the relationship between geometric parameters and coupled physiological behavior. In particular, the combined effect of leaflet height and radius has not been systematically studied under a consistent structural assumption. Building on these limitations, the present work introduces the aspect ratio (AR), defined by leaflet height and radius, as a meaningful dimensionless parameter for characterizing leaflet geometry by varying the leaflet height while maintaining a constant radius. A hyperelastic shell model within an FSI framework is then adopted to systematically investigate its influence on both mechanical and hemodynamic responses, providing a quantitative basis for prosthetic leaflet geometry optimization.

Three-dimensional high-fidelity FSI simulations are necessary to reflect the true mechanical properties of AVs undergoing periodic large deformations. However, these simulations still face numerous significant challenges. (i) Mesh distortions occur when solid elements are used to model highly deformed valve structures, thereby severely compromising computational stability and accuracy. (ii) Excessive simplification of boundary conditions can cause significant deviations from physiological reality, whereas inconsistency between the prescribed inlet and outlet conditions often leads to computational failure. (iii) Achieving physiologically and physically appropriate initialization for numerical simulations remains a critical challenge. Although common practice involves initiating computations from a state of zero velocity, stress and deformation, this approach often fails to account for the inherent physiological pre-stress and initial flow conditions, potentially leading to unrealistic transient behaviors or numerical instabilities during the initial cycles.

To address these challenges, we integrate several computational approaches to yield more physiologically realistic results. The ALE method is adopted to handle the complex coupling between highly deformed valves and the surrounding blood flow. Specifically, within this ALE-based FSI framework, the valve leaflets are discretized using hyperelastic shell elements to achieve accurate and stable descriptions of valve kinematics over multiple cardiac cycles. To further enhance the physiological realism of the simulation, sophisticated boundary conditions are implemented: a velocity profile derived from Doppler imaging data [45] is prescribed at the inlet to replicate patient-specific inflow, while the Windkessel model is employed to determine the outlet pressure by accounting for the impedance effects of the downstream vessel [46]. Moreover, a periodicity-convergent initial state is determined and employed in the present computations to account for the physiological pre-stress and initial flow conditions. Building on the above attempts, we obtain three-dimensional numerical results of the interactions between hemodynamics and valve deformation for multiple complete cardiac cycles. The effects of geometric features, defined as the ratio of leaflet height to radius, are subsequently explored through the analysis of key indicators, such as the GOA, TPG and WSS.

## Materials and methods

### Geometric model

In this study, we refer to the three-dimensional geometry of the AV proposed in the experimental report by Motta et al. [47] and construct three valve leaflet models featuring shell structure in accordance with previous studies [48, 49]. The Mindlin–Reissner plate theory is employed to account for stretching, bending and transverse shear effects [50]. The bottom surface of each valve leaflet is set as the reference surface for the shell formulation with a thickness of 0.55 mm [51]. As illustrated by the cross-sectional diagram in Figure 1, the red dashed line denotes the bottom surface, and the red arrow indicates the thickness direction. The leaflet diameter is fixed at 26 mm [52]. We define the AR as the ratio of the valve height to its radius. On the basis of three different height values that fall within the design range of previous studies [43, 53], the ARs of the three valve models are 0.86, 1.06 and 1.26. The specific geometric parameters are shown in Table 1. For the corresponding leaflet shell models, the surface near the inlet is taken as the bottom surface (i.e. the reference surface of the shell structure), as shown in Figure 2. The geometry of the fluid domain employs a tubular model with a constant diameter equal to the inner diameter of the valve leaflet, while the aortic sinus is modeled as a spherical cap structure [54]. Figure 3 shows the model dimensions.

**Figure 1:**
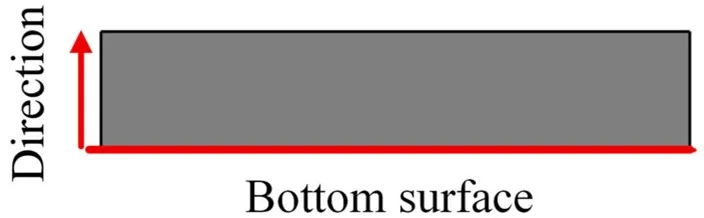
Position and orientation of the shell cross-section.

**Figure 2:**
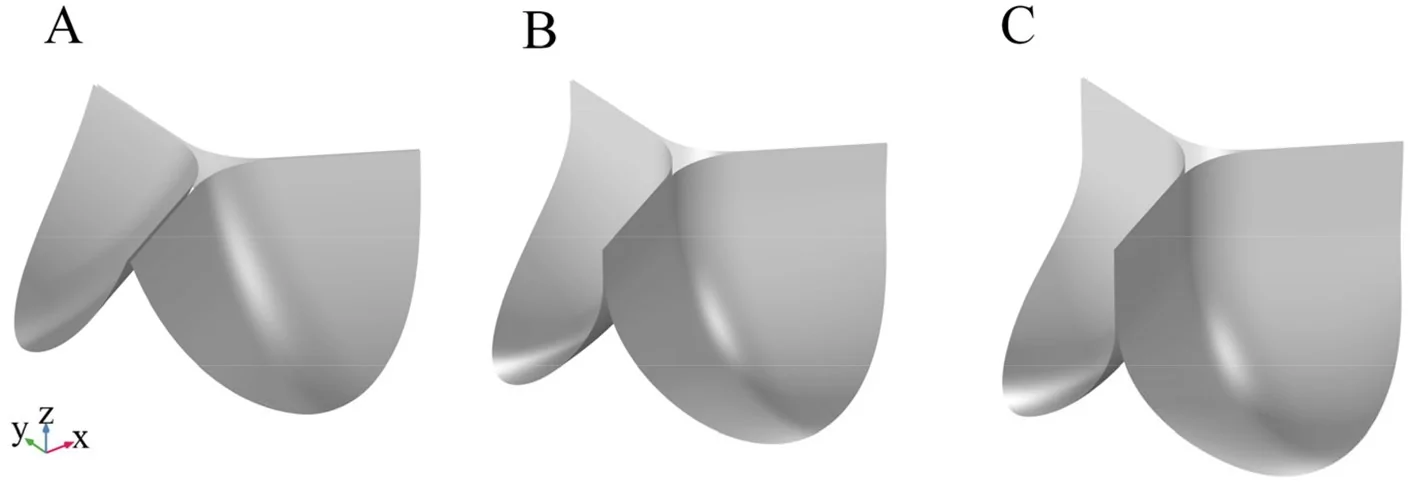
Valve geometric models: (**A**) AR = 0.86, (**B**) AR = 1.06, (**C**) AR = 1.26.

**Figure 3:**
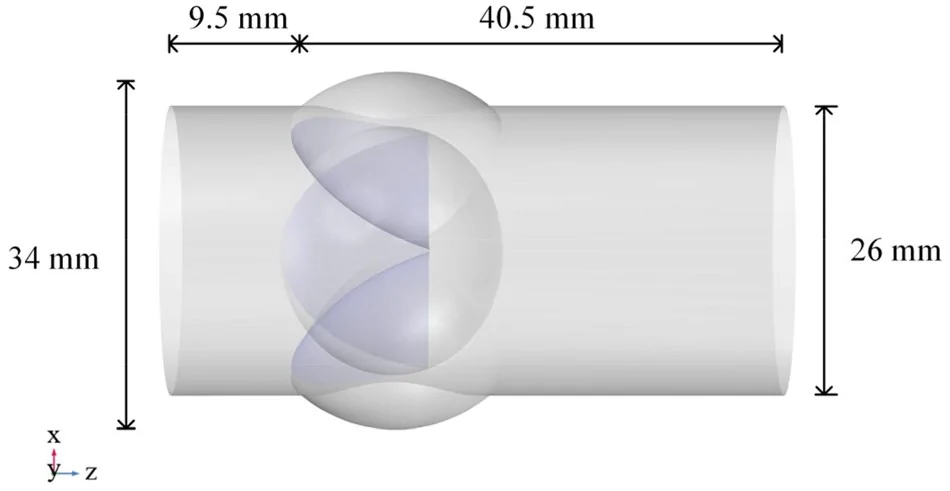
Geometry of a valved vascular conduit.

**Table 1:** Geometric parameters of the valves.

Valve	Valve height (mm)	Aspect ratio
A	11.18	0.86
B	13.78	1.06
C	16.38	1.26

### Governing equations and material

Blood is considered an incompressible unsteady fluid that exhibits strong non-Newtonian behavior under low shear stress. Although some studies have suggested that transitional or turbulent flow structures may arise locally in cardiovascular flows, physiological blood flow under strongly pulsatile conditions is still generally considered laminar [55], as the combined effects of blood velocity, vessel diameter and blood viscosity usually maintain the Reynolds number below the classical transition threshold of approximately 2300 [56]. Previous studies have established that the laminar flow assumption can adequately capture the main hemodynamic characteristics while maintaining computational stability and efficiency [23, 27, 29, 31]. Therefore, a laminar flow model is adopted in this study. In the ALE-based FSI model, the fluid domain is adapted to the motion of the valve via the moving meshing technique [57–59]. In this study, both the Newtonian fluid model and the Carreau–Yasuda non-Newtonian fluid model are employed in practical computations. The continuity and momentum equations are expressed as follows:


(1) $$\nabla \cdot v_{f}=0$$

(2) $$\rho_{f}\frac{\partial v_{f}}{\partial t}+\rho_{f}\left((v_{f}-v_{g})\cdot \nabla \right)v_{f}=-\nabla p+\nabla \cdot \left[\mu \left(\nabla v_{f}+{\left(\nabla v_{f}\right)}^{T}\right)\right]$$

where $v_{f}$ is the blood flow velocity, $\rho_{f}$ is the density of blood flow, $v_{g}$ is the grid velocity, p is pressure and *μ* is the blood viscosity coefficient. For a Newtonian fluid, *μ* is a constant, whereas the viscosity of a non-Newtonian fluid is governed by


(3) $$\mu \left(\gamma \right)=\mu_{\infty }+\left(\mu_{0}-\mu_{\infty }\right){\left[1+{\left(\lambda \gamma \right)}^{m}\right]}^{\frac{n-1}{m}}$$

Here, *γ* denotes the shear rate, $\mu_{\infty }$ is the viscosity at an infinite shear rate, $\mu_{0}$ is the viscosity at zero shear rate, *λ* denotes the relaxation time and *m* and *n* represent the transition parameter and the power exponent, respectively.

The solid domain includes the AV and the vessel with the sinus. Marom et al. [52] and Kivi et al. [34] reported that the effect of compliant and rigid aorta settings on leaflet motion is negligible. Therefore, the vessel in this study is modeled as a rigid wall. With respect to valve leaflets, Sacks et al. verified the near-incompressibility of biological valves via biaxial mechanical testing [60]. Although the valve is physiologically anisotropic, implementing a fully anisotropic model is still highly challenging, and previous studies have shown that the isotropic assumption under low-strain conditions is reasonable [18, 19, 61]. Accordingly, the valve is treated as an isotropic, hyperelastic and nearly incompressible material in the present computations. The displacement $u_{s}$ at any point within the shell can be expressed in terms of the bottom-surface displacement and the rotational displacement. The deformation gradient tensor ***F*** is constructed from $u_{s}$ as follows:


(4) $$u_{s}=u_{\text{shell}}+zh\mathrm{ar},z\in \left[0,1\right]$$

(5) $$F=I+\nabla u_{s}$$

where $u_{\mathrm{shell}}$ is the displacement of the shell bottom surface, *h* is the leaflet thickness, ***a*** is the unit normal vector of the shell bottom surface, ***r*** is the rotation vector of the surface normal and *z* is the normalized thickness coordinate. The constitutive response of the valve is governed by the Neo–Hookean strain energy function


(6) $$W=\frac{1}{2}G\left(I_{1}-3\right)+\frac{1}{2}K{\left(J-1\right)}^{2}$$

*I*_1_ is the first strain invariant, and *J* is the square root of the determinant of the right Cauchy–Green deformation tensor ***C***. *G* and *K* represent the shear modulus and the bulk modulus, respectively. Based on the strain energy function, the second Piola–Kirchhoff stress tensor ***S*** can be obtained by taking the derivative with respect to ***C***.


(7) $$S=2\frac{\partial W}{\partial C}$$

Finally, the dynamic motion of the leaflet is governed by the momentum equation of the solid structure


(8) $$\rho_{s}\frac{\partial^{2}u_{s}}{\partial t^{2}}-\nabla \cdot {\left(\mathrm{FS}\right)}^{T}-f_{V}^{s}=0$$

where $\rho_{s}$ is the density of the valve and $f_{v}^{s}$ denotes the equivalent structural loading induced by the traction forces at the fluid–structure interface.

Notably, during the closing phase, the valve leaflets undergo rapid deformation due to pressure differences. To account for tissue damping effects, the damping stress is defined by Marom et al. [52] as follows:


(9) $$\sigma_{d}=\beta \cdot D_{\text{el}}\cdot \varepsilon$$

Here, $\sigma_{d}$ is the additional damping stress, *β* is the stiffness proportionality coefficient (viscoelastic damping coefficient), ***D***_el_ is the elastic stiffness and *ε* is the strain rate. Referring to the damping coefficient sensitivity study of Marom et al. [52], the stiffness proportion damping coefficient of the valve is set to 0.01 s. After finite element-based discretization, the above damping stress formulation corresponds to the stiffness-proportional component of Rayleigh damping [52], and the damping matrix in the structural dynamic equation can be written as follows:


(10) H=α⋅M+β⋅N

where ***H*** is the structural damping matrix. ***M*** is the structural mass matrix. ***N*** is the structural stiffness matrix. In Equation (10), the mass proportionality coefficient *α* is set to zero, and the damping effect is governed by the stiffness-proportional damping term, β⋅N.

Table 2 lists the material property values for blood and valve leaflets [23, 61, 62]. When blood is non-Newtonian, the following parameters are used in the Carreau–Yasuda model [62]: $\mu_{\infty }=0.0035\;\text{Pa}\cdot s$, $\mu_{0}=0.056\;\text{Pa}\cdot s$, λ=8.2 s, a=0.64 and n=0.2128.

**Table 2:** Material properties of the blood and valve.

Parameters	Value
Newtonian blood viscosity (Pa·s)	0.0035
Blood density (kg/m^3^)	1050
Valve density (kg/m^3^)	1200
Valve shear modulus (MPa)	1.50
Valve bulk modulus (MPa)	150
Valve Poisson’s ratio	0.495

### Boundary conditions

Fixed constraints are applied to the entire lower edge of the valve to reproduce the fixation of the annulus by a rigid stent in artificial valve design practice, while the tip edge of the valve is designed as a free edge [63]. During the valve closure phase, contact behavior between the leaflets must be prescribed. Since the bottom surface is used as the reference surface for the shell design in this study (see Figure 1), the contact pairs among the three leaflets are defined directly on these bottom surfaces, with each leaflet alternately serving as the target surface and contact surface relative to the others. The contact offset is therefore set to zero, and the initial gap between the leaflets is specified as 0.1 mm.

At the vessel inlet, we apply a time-varying velocity profile based on Doppler experimental data [45, 64]. Figure 4A shows the velocity curve over a complete cardiac cycle, including the systole (0.00–0.33 s), regurgitation (0.33–0.39 s) and diastole (0.39–0.80 s) stages [27, 65]. To address the pressure reflection effects [55], the Windkessel model, as schematically depicted in Figure 5, is employed to determine the downstream pressure at the outlet [32].


(11) $$Q\left(t\right)=\frac{P_{C}(t)}{R_{d}}+C_{w}\frac{dP_{C}(t)}{dt}$$

(12) $$P\left(t\right)=Q\left(t\right)R_{p}+P_{C}\left(t\right)$$

**Figure 4:**
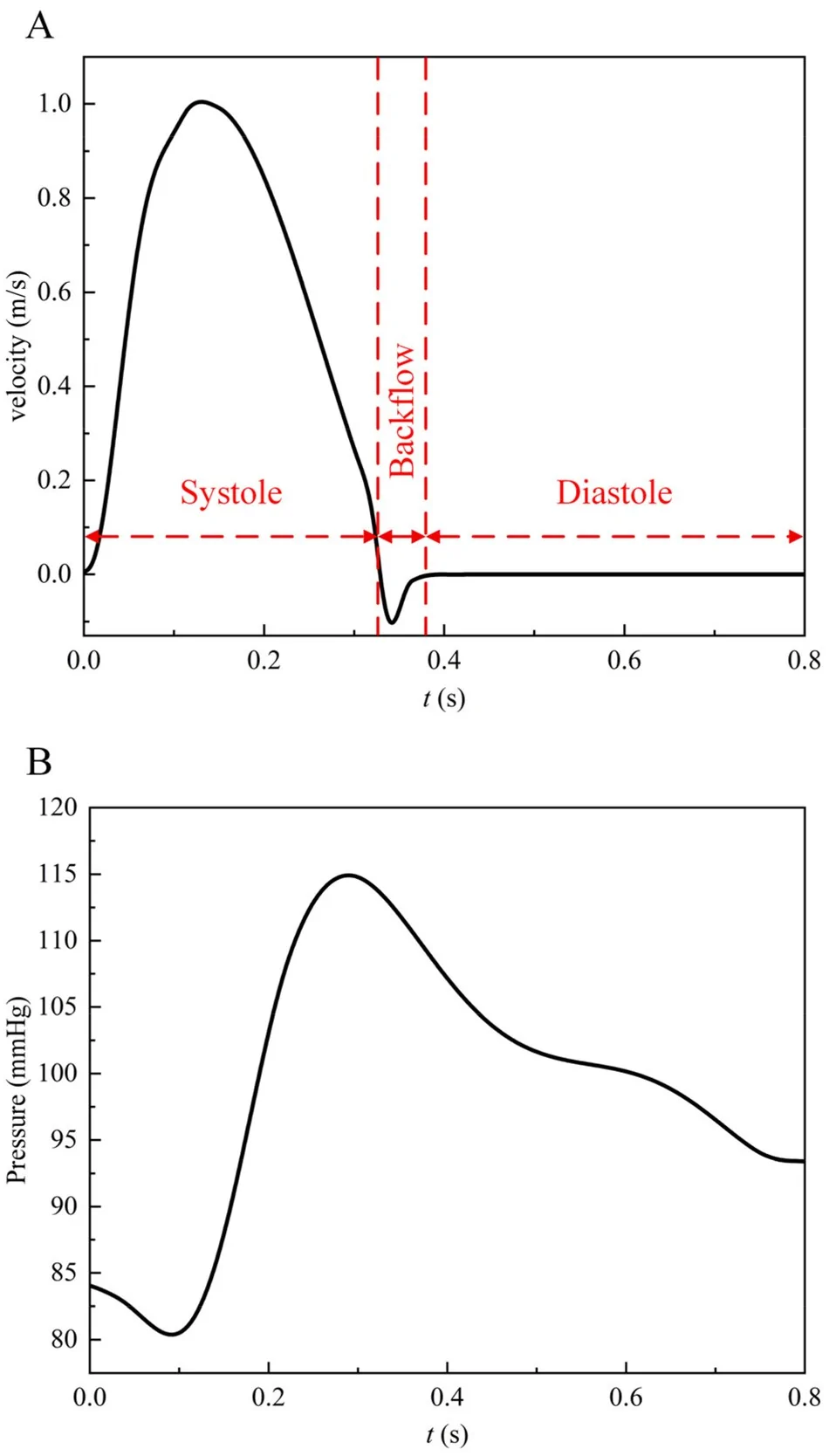
Transient boundary conditions of blood flow: (**A**) inlet velocity, (**B**) outlet pressure.

**Figure 5:**
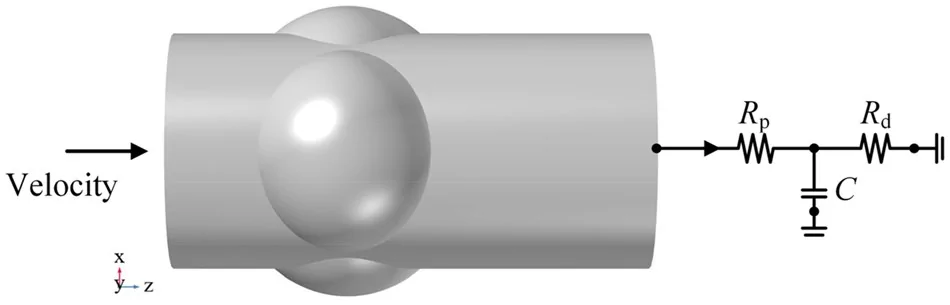
Blood flow direction and schematic diagram of the Windkessel model.

*Q* (*t*) represents blood flow, and *P*(*t*) and *P*_C_(*t*) denote proximal and distal blood pressure, respectively. *R*_p_ is the proximal resistance, *R*_d_ is the distal resistance and *C*_w_ is the total arterial compliance reflecting the elasticity of the vessel [66]. The parameters for the three-element Windkessel model are provided in Table 3 [67]. As shown in Figure 4B, the outlet pressure, derived from the above Windkessel model, ranges from 80 to 120 mmHg, which remains at a physiologically reasonable level and fits the healthy aortic state. The pressure curve predicted here shows good agreement with the results in Ref. [68] and matches the experimental pulsatile flow measurements reported in Ref. [48].

**Table 3:** Parameters for the Windkessel model.

$C_{w}$ (cm^5^/dyne)	$R_{d}$ (dyne·s/cm^5^)	$R_{p}$ (dyne·s/cm^5^)
8e-4	0.17e4	110

Timely FSI is achieved through the following vector constraint conditions at the fluid–structure interface, ensuring compatibility in velocity, displacement and traction force.


(13) $$v_{f}=v_{s}$$

(14) $$u_{f}=u_{s}$$

(15) $$f_{f}=f_{s}$$

Here, ***ν*** is the velocity, ***u*** is the displacement and ***f*** is the traction force with subscripts *f* and *s* representing the fluid and solid, respectively.

### Mesh generation

To achieve more smoothly deformed valve surfaces and capture the valve stress and deformation more accurately, the valve leaflets are discretized using triangular shell elements, with local refinement at the suture line between the leaflet and the vessel. The fluid domain is resolved by tetrahedral elements, and boundary layers are applied to the regions close to both the vascular wall and the leaflet surface. In particular, the vascular wall boundary layer consists of eight layers with a stretching factor of 1.15 and a total thickness of approximately 0.27 mm. The boundary layer on the leaflet surface consists of four layers with a stretching factor of 1.1 and a total thickness of approximately 0.09 mm. Figure 6 shows the meshes for the AV and the fluid domain.

**Figure 6:**
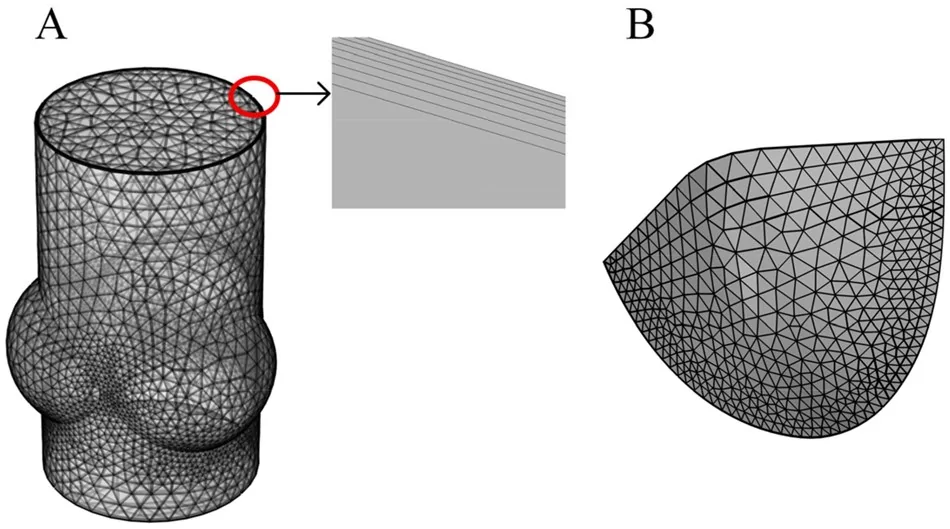
Mesh generation: (**A**) fluid domain mesh with boundary layer, (**B**) aortic valve mesh.

### Numerical solution

In this work, FSI simulations of the valve are performed using the fluid–shell interaction module in COMSOL Multiphysics. The coupled fluid–structure system is solved using a segregated solution approach, in which the fluid variables (velocity and pressure), structural displacement field and spatial mesh displacement field are treated as separate segregated groups. The incompressible Navier–Stokes equations together with the continuity equation in the fluid domain are solved using the finite element method. A standard Galerkin formulation is employed to derive the weak form of the governing equations. Quadratic interpolation is adopted for the velocity field, whereas linear interpolation is used for the pressure field, corresponding to the Taylor–Hood element formulation. Gradients are evaluated through finite element shape functions. The discretized governing equations thereby form a coupled pressure–velocity system for incompressible flow, which is solved using an incremental pressure-correction scheme with iterative updates of the pressure and velocity fields [69]. The structure domain is discretized using the finite element method with quadratic interpolation for the displacement field. The spatial mesh displacement field is updated using the ALE formulation. Within each time step, the velocity and pressure fields, the structural displacement field and the spatial mesh displacement are treated as separate segregated steps and are iteratively updated until the nonlinear solution converges. The nonlinear iterations are performed using the Constant (Newton) method. For the segregated fluid step, the resulting linear systems are solved using an algebraic multigrid iterative solver, whereas the remaining segregated steps, including the structural displacement field and the spatial mesh displacement, are solved using direct solvers. Nonlinear iterations at each time step are considered converged when the relative residual falls below 10^−4^. Within the ALE framework, an automatic mesh smoothing and remeshing strategy is employed in the fluid domain to accommodate valve leaflet deformations. When the mesh distortion metric exceeds a prescribed threshold (set to 2 in this study), remeshing is triggered to regenerate high-quality meshes. Below the prescribed threshold, remeshing is not activated, and the fluid mesh is updated solely through the ALE moving mesh. Temporal integration is carried out using the implicit backward differentiation formula (BDF) scheme. A baseline time step size of 0.001 s is adopted for all simulations.

To ensure numerical accuracy and solution independence, a systematic verification procedure is conducted. (i) Four cardiac cycles are simulated using the valve model shown in Figure 2A (AR = 0.86) to ensure periodicity–convergence and determine the physiologically appropriate initial state accounting for the pre-stress and initial flow conditions. The results indicate that from the second cardiac cycle onward, the flow field and structural responses exhibit good periodic consistency (see Supplementary Data A). (ii) A grid independence study is conducted for all three valve models (AR = 0.86, 1.06 and 1.26) by comparing results from three mesh resolutions (coarse, medium and fine). The medium mesh is selected for subsequent simulations due to its favorable balance between numerical accuracy and computational cost (see Supplementary Data B). (iii) The time step sensitivity is evaluated for the valve model with AR = 0.86 by increasing the time step size to 0.005 s while keeping all the other numerical settings unchanged. The results show that a time step of 0.005 s has a relatively small effect on the leaflet stress and overall deformation. However, a larger time step may reduce the ability to capture transient flow details in the flow field. Therefore, a time step of 0.001 s is adopted to ensure sufficient temporal resolution and numerical accuracy (see Supplementary Data C). (iv) The numerical model is validated against the study by Liu et al. [37] by adopting the same computational framework, including the valve geometry, constitutive model, material parameters, FSI formulation and boundary conditions. The simulated leaflet deformation and hemodynamic characteristics show good agreement with the published results, demonstrating the accuracy and reliability of the present numerical framework (see Supplementary Data D).

### Postprocessing

Postprocessing of the computation results is necessary to achieve insightful evaluation of the valve mechanical properties and hemodynamics.

- The GOA serves as a crucial indicator for assessing the opening and closing functions of artificial valves [51]. As illustrated in Figure 7, the valve leaflets reach their maximum deformation state, and their outer surfaces projected onto the XY plane. We note that this projection may produce overlapping regions because of the greater leaflet deformation. To address this issue, the leaflet free edges are identified to obtain the geometric orifice surface for the GOA calculation. For the asymmetric deformation configuration, the orifice surface is reconstructed by extracting the leaflet-free edges using the same procedure described above.
- The TPG represents the average difference in pressure across the AV during the systolic phase (0.00–0.33 s), indicating energy loss. Its definition is as follows [32]:
  (16) $$\mathrm{TPG}=\frac{1}{T}\int_{T}\Delta Pdt$$
  where Δ*P* denotes the difference in pressure between the aortic side and the left ventricular side. *T* is the systolic duration.
- The RF reflects the ratio of regurgitant blood volume through the AV to the total blood volume and is defined as follows [32]:
  (17) $$\mathrm{RF}=\frac{\left|\text{TSV}-\text{FSV}\right|}{\text{FSV}}$$
  where TSV and FSV denote the total blood volume and forward blood flow volume, respectively.
- The WSS is used to characterize the tangential stress at the wall produced by the velocity gradient of viscous blood flow and is regarded as one of the most important hemodynamic indicators reflecting the interaction between blood flow and the wall [70]. In this study, the WSS is calculated to quantitatively evaluate the local impingement and shear-loading regions on the valve surfaces, as defined in Equation (18). In addition, the AWSS of the cardiac valves is employed to characterize the spatial average of the WSS exerted upon the valve surface and is obtained by Equation (19):
  (18) $$\mathrm{WSS}=\left|\mu \frac{\partial v_{f}}{\partial \eta }\right|$$
   
  (19) $$\mathrm{AWSS}=\frac{\sum {\text{WSS}}_{i}\cdot {\text{area}}_{i}}{\sum {\text{area}}_{i}}$$

**Figure 7:**
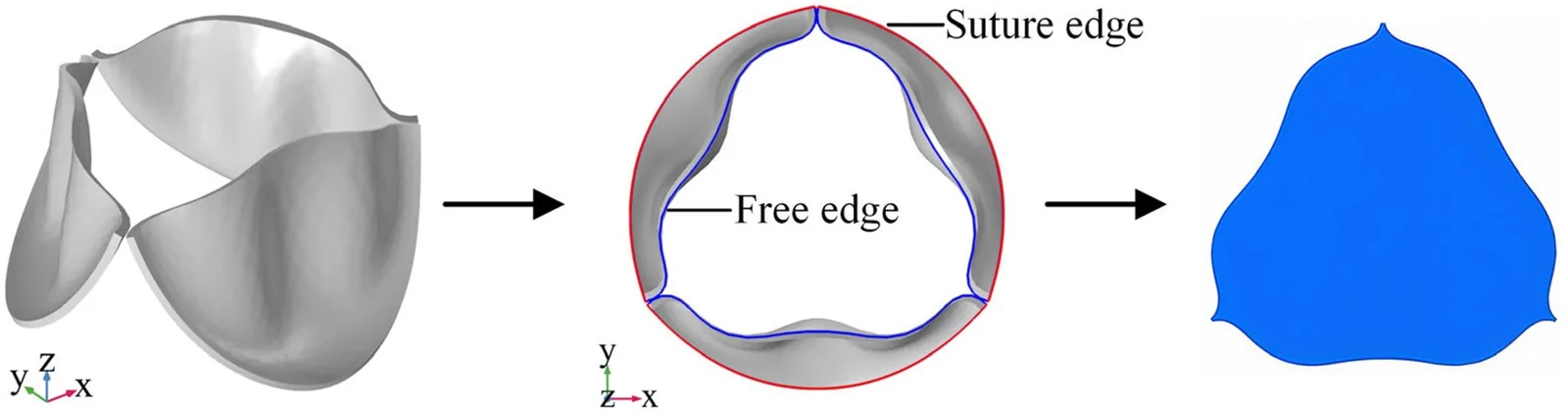
Flowchart of GOA calculation.

where $\frac{\partial v_{f}}{\partial \eta }$ is the gradient of the blood velocity vector, with *η* being the wall-normal coordinate. ${\text{WSS}}_{i}$ is the WSS of element *i*, and ${\text{area}}_{i}$ represents the area of element *i*.

## Results and discussion

### Effects of AR on aortic valve dynamics

The geometric features of valve leaflets significantly affect mechanical performance metrics such as leaflet stress and the GOA [43, 47, 53]. Therefore, in this section, the influence of the AR (0.86, 1.06 and 1.26), which reflects the combined effects of the valve radius and height, on both mechanical behavior and hemodynamic performance is investigated through FSI simulation.

Figure 8 illustrates the blood velocity distributions and streamlines on the XZ plane at different times for various AR cases. For all AR cases, the overall evolution trend of the flow velocity magnitude on the XZ cross-section is consistent with the experimental observations [9] and the numerical results [23]. Specifically, during early systole (0.00–0.14 s), the magnitude of the blood velocity gradually increases and peaks at *t *= 0.14 s. Subsequently, the magnitude of the blood velocity progressively decreases. At *t *= 0.34 s, the flow enters the regurgitation phase, characterized by reverse blood motion through the valve, after which the flow transitions into the diastole stage.

**Figure 8:**
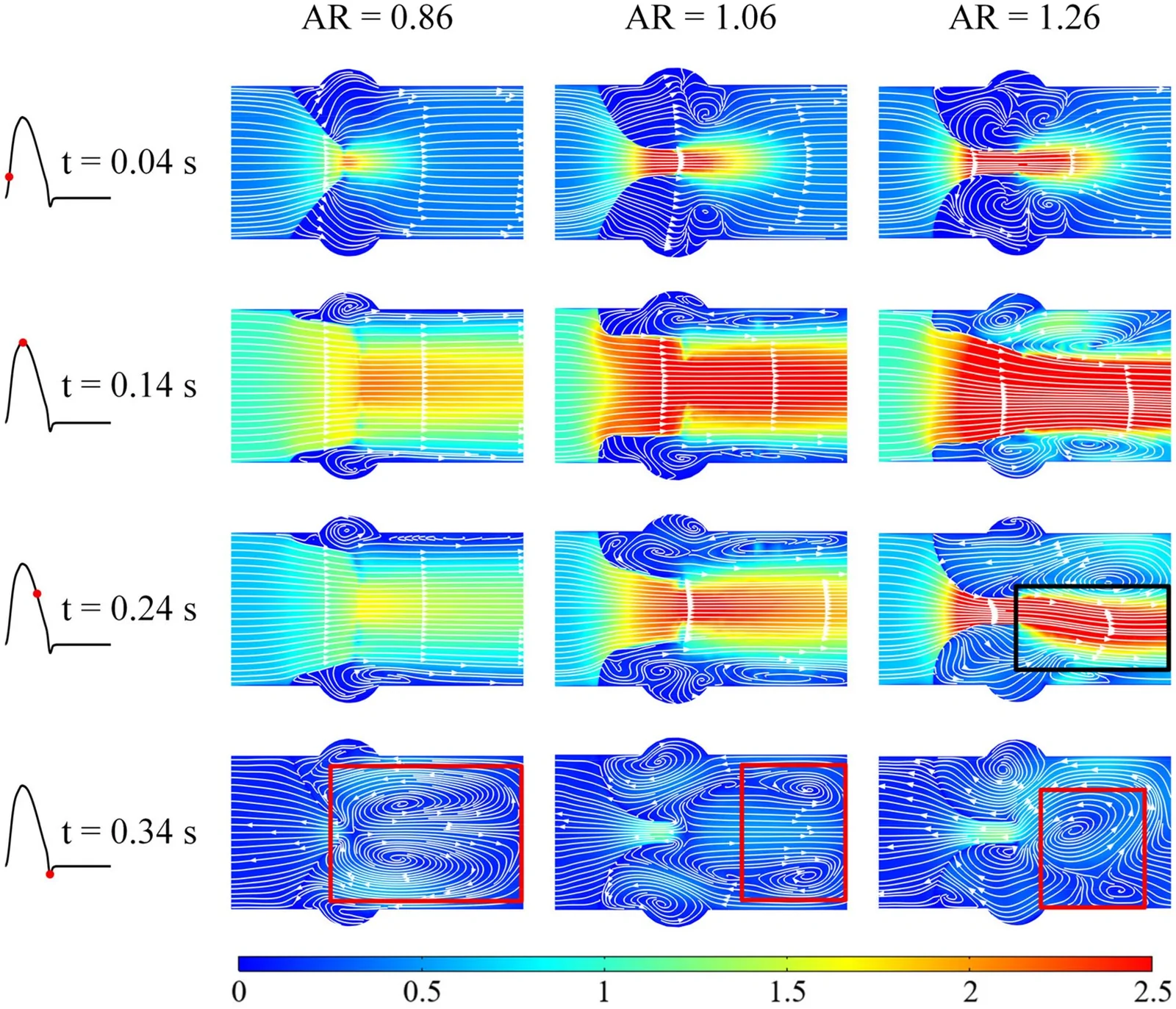
Velocity magnitude (m/s) distribution and streamlines on the XZ cross-section.

At early systole (*t *= 0.04 s), noticeable differences in the flow fields emerge as the AR increases. At AR = 0.86, the sinus region maintains a relatively regular laminar flow pattern. When the AR increases to 1.06, a distinct vortex begins to form at the junction between the sinus and the downstream vessel wall. With further progression to AR = 1.26, more complex flow structures develop, with vortices present both within the sinus and at its junction with the downstream vessel wall, indicating an increasing level of flow disturbance associated with higher AR values.

When the blood flow velocity reaches its peak at *t *= 0.14 s, strong jet flow occurs in all the cases, and a vortex structure emerges within each sinus. For cases with lower AR values, the jet is generally well aligned with the centerline, and the flow structure roughly maintains symmetry with respect to the centerline. However, symmetry breaks at AR = 1.26. Slight deviations in the jet flow are observed, shifting toward the lower side of the cross-sectional plane. In addition, the magnitude of the blood velocity increases notably for cases with greater ARs. Quantitatively, compared with the AR = 0.86 case, whose maximum velocity is 2.14 m/s, the peak velocities for AR = 1.06 and AR = 1.26 significantly increase to 3.04 and 5.22 m/s, respectively, corresponding to approximately 1.42 and 2.44 times increases, respectively.

By *t *= 0.24 s, the flow velocity magnitude decreases markedly; thus, the strength of the jet flow gradually weakens in all the cases. Downstream of the vessel, the laminar flow of the AR = 0.86 case in the near-wall region only undergoes slight deflections, indicating a tendency to form vortices. However, for AR = 1.06 and 1.26, distinct vortices have already formed since *t *= 0.14 s. Notably, in the AR = 1.26 case, the jet deviation becomes pronounced (indicated by the black box in Figure 8). Concurrently, the scale differences of the two vortices in the downstream near-wall region become increasingly significant.

Upon entering the backflow phase (*t *= 0.34 s), the blood flow in the near-wall region downstream of the vessel forms wide-ranging vortices that progressively move away from the vessel wall. Moreover, a smaller-scale reverse jet is generated. Under these flow conditions, clear differences in the downstream flow patterns emerge among the different AR cases (as indicated by the red boxes in Figure 8). At AR = 0.86, the downstream flow field remains largely symmetric. As the AR increases to 1.06, the overall symmetry is still preserved, although the vortical structures are slightly shifted toward the outlet. In contrast, at AR = 1.26, the downstream flow field completely loses symmetry and regularity.

When blood flow consistently deviates toward the lower side of the vessel wall in the XZ-plane, the deflected jet continuously impinges on the lower wall, thereby maintaining elevated hemodynamic loading over the affected region of the vessel wall. As the flow continues to develop, the deviation of the jet from the vessel centerline alters the downstream flow pattern, resulting in the formation of asymmetric vortex structures.

The deformed configuration and von Mises stress distribution of the valve leaflets are shown in Figure 9, and the deformation behavior of the valve is generally consistent with the experimental observations by Sigüenza et al. [71] and Liu et al. [48]. During the systolic phase (0.00–0.33 s), the von Mises stress is primarily concentrated on the suture edges and in the belly region of the valve. In the early systole stage (0.00–0.14 s), the increase in blood flow velocity promotes the opening of the leaflets. At this stage, notable differences in the GOA among the various cases are observed: a larger AR leads to a smaller GOA. When the blood flow velocity reaches its peak (*t *= 0.14 s), both the stress and the GOA reach their maximum values. The deformation configuration of the leaflets remains symmetrical for three cases with AR = 0.86, 1.06 and 1.26 exhibits an asymmetrical deformation pattern, resulting in an uneven stress distribution. This behavior corresponds to its asymmetric jet flow structure (see Figure 8). At late systole (*t *= 0.24 s), blood flow decreases, and the leaflets gradually close. However, the valve with AR = 1.26 is almost fully closed at this point, leading to a lower systolic flow volume than that of the other two models.

**Figure 9:**
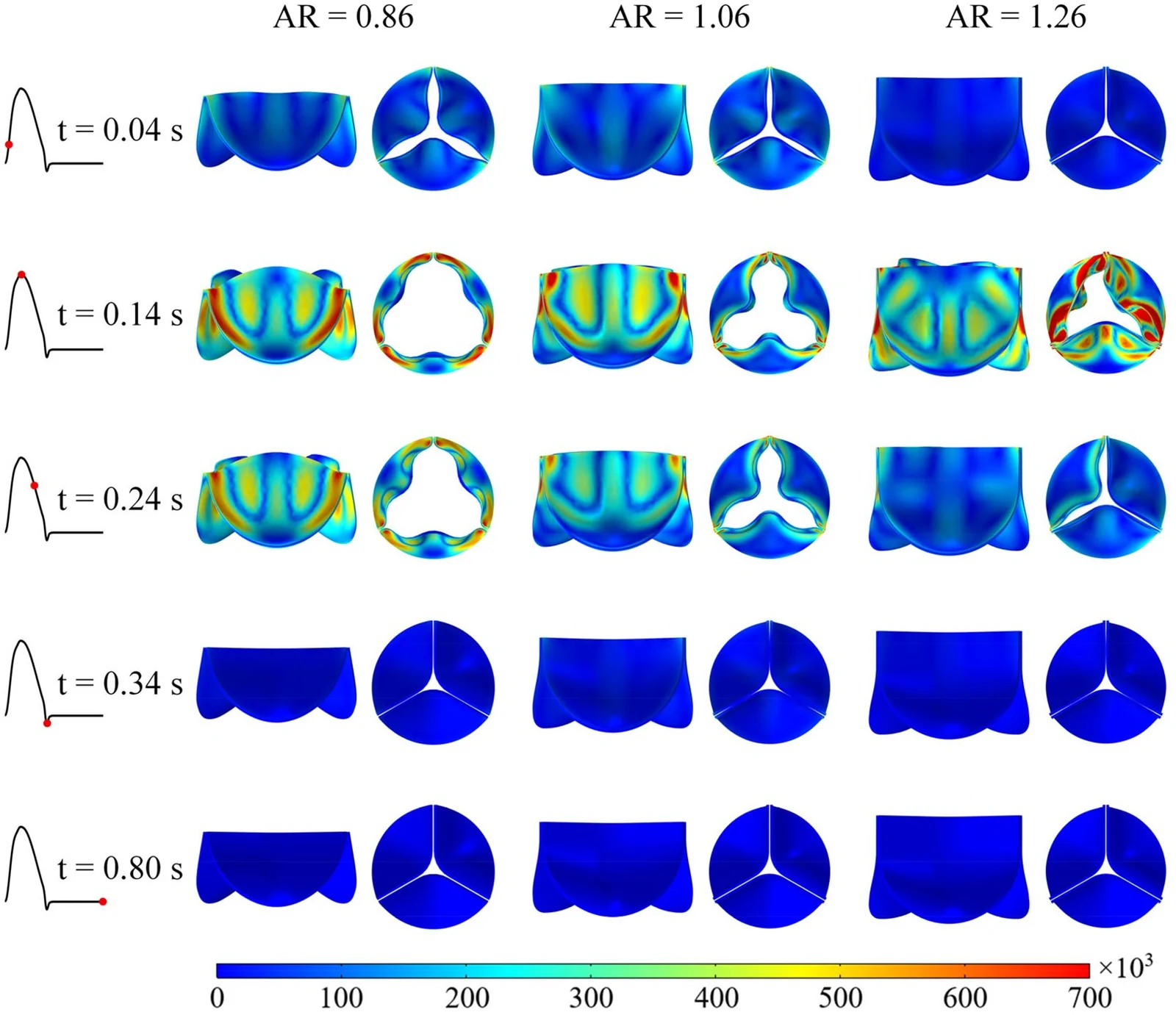
Von mises stress (Pa) distribution and deformed configuration of the valve leaflets.

Table 4 presents the maximum von Mises stress (*σ*_Vmax_), the maximum GOA (GOA_max_) and the TPG over the systole (0.00–0.33 s) for three valve models with different ARs. For the AR = 0.86 case, the present results are in good agreement with those of previous studies using solid element models. Specifically, Oks et al. [27] reported a maximum GOA of 200–300 mm², Labrosse et al. [72] reported a maximum stress value of approximately 840 kPa, and Costa et al. [61] reported a peak TPG of approximately 12 mmHg. Moreover, the results of the present analysis indicate that with increasing AR, the maximum von Mises stress increases while the GOA decreases, which is completely opposite to the findings from pure structural mechanics analysis [43]. Since the TPG is calculated by time averaging the instantaneous pressure difference Δ*P* over the systolic phase according to Equation (16), the variation of Δ*P* over time is further illustrated in Figure 10. The results show that Δ*P* increases for higher AR, and the pressure difference is substantially greater for the valve with AR = 1.26 than the other two cases throughout systole. According to the ISO-5840 international standard [73], the effective orifice area (EOA) of an AV with a radius of 13 mm should be larger than 145 mm², whereas the GOA of the valve with an AR of 1.26 is 83.03 mm², and the TPG reaches as high as 105.12 mmHg. Furthermore, the RF increases significantly with increasing AR: the RF values corresponding to the three AR = 0.86, 1.06 and 1.26 cases are 0.1%, 5.86% and 19.07%, respectively. The RF of 19.07% at AR = 1.26 indicates that, in addition to stenosis risk, the valve also exhibits regurgitation.

**Figure 10:**
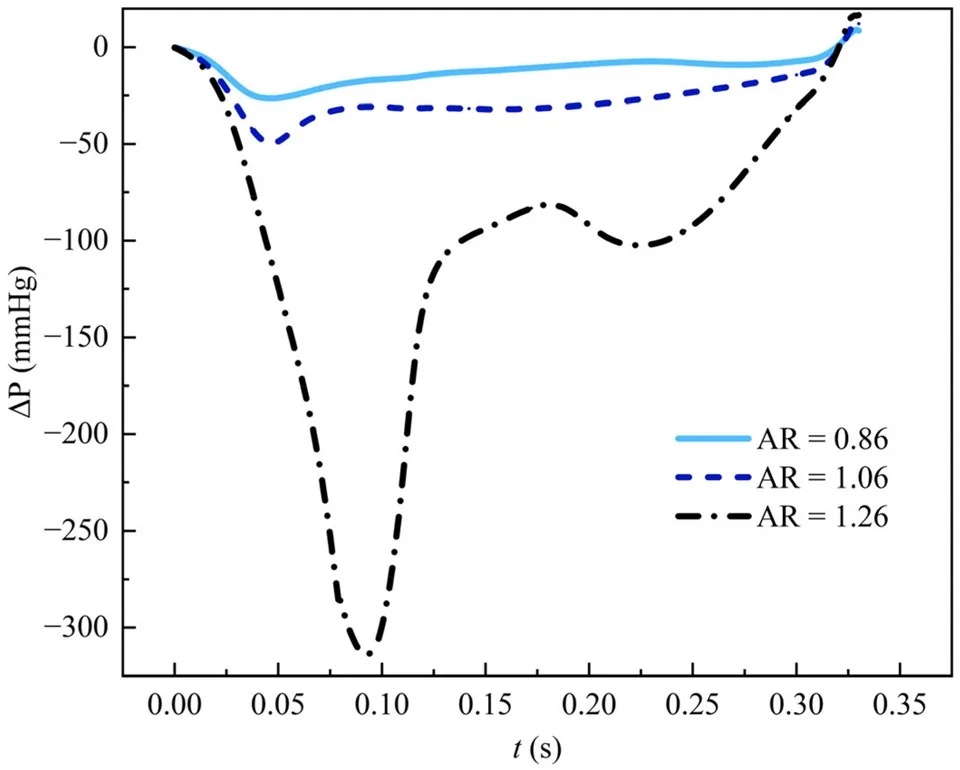
Curves of the difference in pressure between the aortic side and the left ventricular side.

**Table 4:** Maximum von mises stress, maximum GOA and TPG at different ARs.

AR	$\sigma_{V}{\;}_{\max}$ (kPa)	${\text{GOA}}_{\max}$ (mm^2^)	TPG (mmHg)
0.86	762	291.74	11.80
1.06	953	175.67	26.17
1.26	1209	83.03	105.12

WSS is distributed on the ventricular side and aortic side surfaces of the valve [11], and endothelial damage to the valve is associated with elevated WSS. Figure 11 shows the instantaneous WSS distribution at *t *= 0.14 s. As the AR increases, the overall WSS level exhibits an upward trend, while distinct spatial patterns of high-WSS regions are observed between the ventricular side and aortic side surfaces. On the ventricular side, high-WSS regions are primarily concentrated in the leaflet curved belly under low AR and gradually extend toward the tip regions of the valve as the AR increases (see Figure 11A). In contrast, on the aortic side, it is concentrated at the leaflet tip under low AR and subsequently expands to the curved belly regions with increasing AR (see Figure 11B).

**Figure 11:**
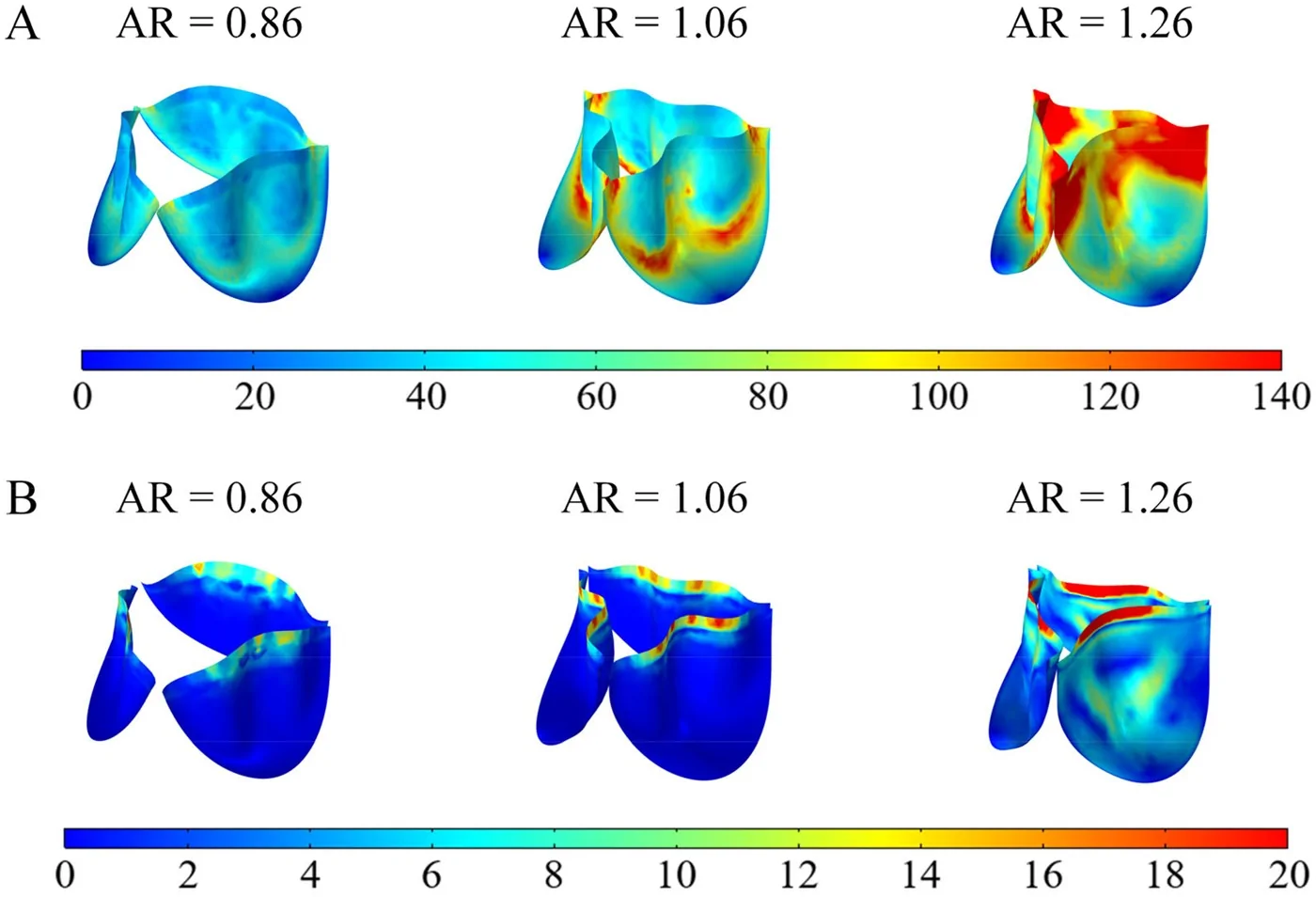
WSS (Pa) Distribution at peak blood flow: (**A**) the ventricular side, (**B**) the aortic side.

The area-averaged WSS on the valve during systole is calculated, yielding the AWSS. Figure 12A shows the evolution of the AWSS on the ventricular side of the valve. As the AR increases, the AWSS tends to increase. Notably, when AR = 1.26, the AWSS is significantly greater than that of the other two groups, reaching a maximum of 218.80 Pa. Figure 12B shows the variation in the AWSS on the aortic side of the valve. Although the AWSS on this surface is significantly lower than that on the ventricular side, it reaches its highest value (16.08 Pa) at AR = 1.26. Notably, during the blood acceleration phase (0.00–0.14 s), the AWSS for AR = 0.86 is greater than that for AR = 1.06. This behavior is contrary to the general trend observed on the ventricular side, where the AWSS increases with increasing AR. This phenomenon can be interpreted based on Equation (19) and is associated with the extent of the high-WSS region. As shown in Figure 11B, the spatial distribution of the high-WSS region for AR = 0.86 is larger than that for AR = 1.06 on the aortic side.

**Figure 12:**
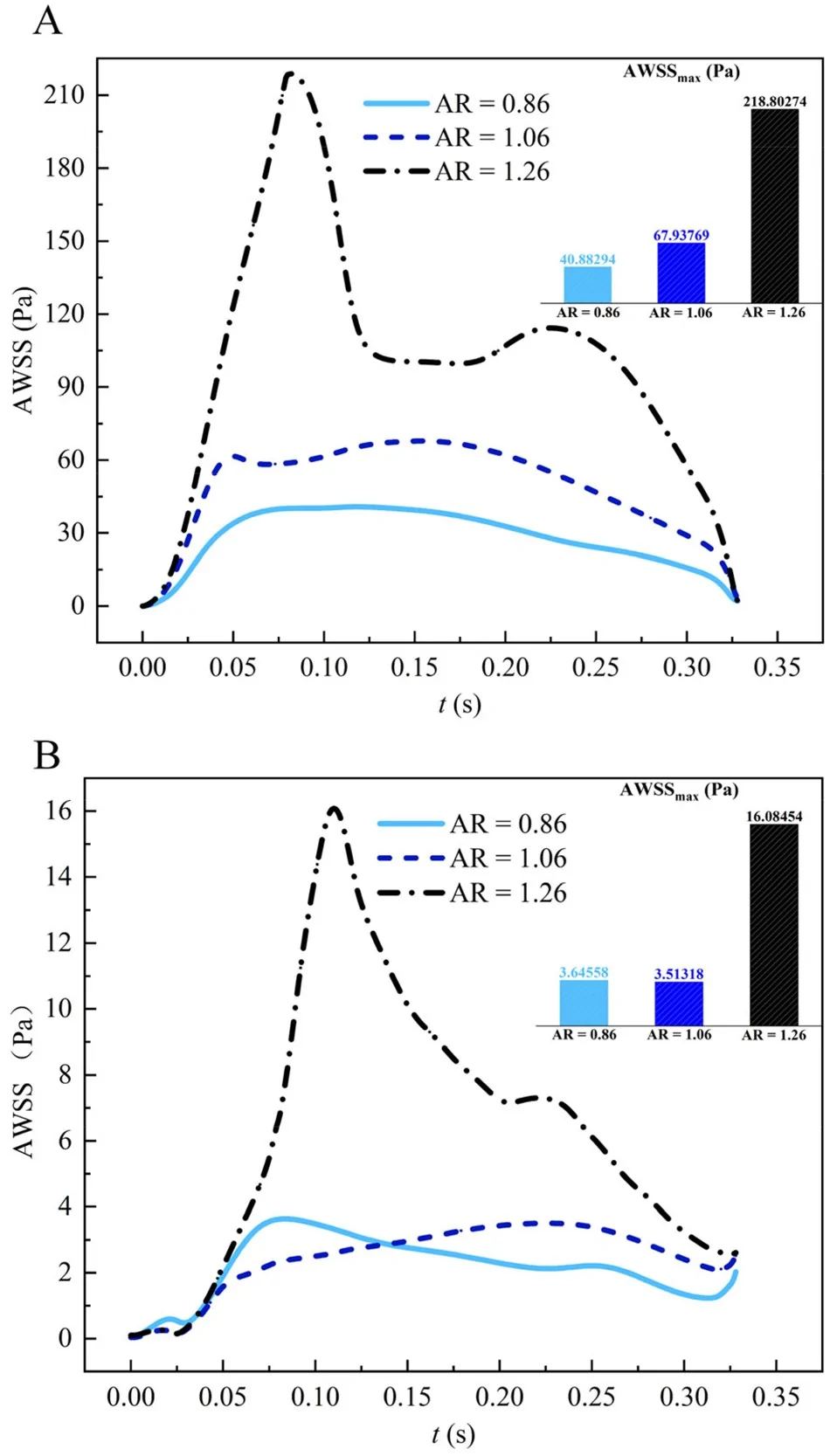
AWSS During systole on (**A**) the ventricular side and (**B**) the aortic side, along with bar charts showing the maximum AWSS values.

In this section, the mechanical and hemodynamic properties of valves with various ARs (AR = 0.86, 1.06 and 1.26) are compared. The results indicate that as the AR increases, the valve performance systematically deteriorates: The valve with AR = 1.26 has the smallest GOA (83.03 mm²), the highest TPG (105.12 mmHg) and the greatest RF (19.07%). Moreover, this model exhibits significant jet deflection during systole, asymmetric leaflet deformation behavior and uneven stress distribution, indicating valvular pathology.

To further clarify the underlying mechanism responsible for the distinct behavior observed at AR = 1.26, an additional case with AR = 1.16 is investigated. The results demonstrate that increasing the AR from 1.06 to 1.16 further intensifies the mechanical loading and hemodynamic alterations. At AR = 1.16, the maximum leaflet von Mises stress increases to 1056 kPa, accompanied by a reduction in GOA to 119.15 mm², representing a 32.17% decrease compared with the AR = 1.06 case (175.67 mm²). Correspondingly, the decreased GOA restricts blood flow passage, resulting in an increase in peak velocity magnitude to 4.12 m/s, which is 35.5% higher than that of the AR = 1.06 case (3.04 m/s), and consequently elevates the TPG to 53.79 mmHg. The leaflet motion remains relatively symmetric at AR = 1.16 (see Figure 13), and no apparent jet deviation is observed in the velocity magnitude distribution (see Figure 14). Nevertheless, the unequal size of vortical structures near the two sides of the vessel wall suggests that the flow field has begun to exhibit asymmetric characteristics, indicating a potential for jet deviation. Figure 15 presents the AWSS curves on the ventricular and aortic surfaces of the leaflets for the cases with AR = 1.06, 1.16, and 1.26. The results indicate that AWSS gradually increases with increasing AR on both surfaces. For the AR = 1.16 case, the maximum AWSS is 107.91 Pa on the ventricular side and 6.55 Pa on the aortic side. The AR = 1.16 case further confirms that increasing AR progressively restricts leaflet deformation and reduces the GOA. The reduced GOA accelerates blood flow and enhances convective effects, thereby making the flow field more prone to asymmetric patterns and indicating a transition toward deteriorated valve performance.

**Figure 13:**
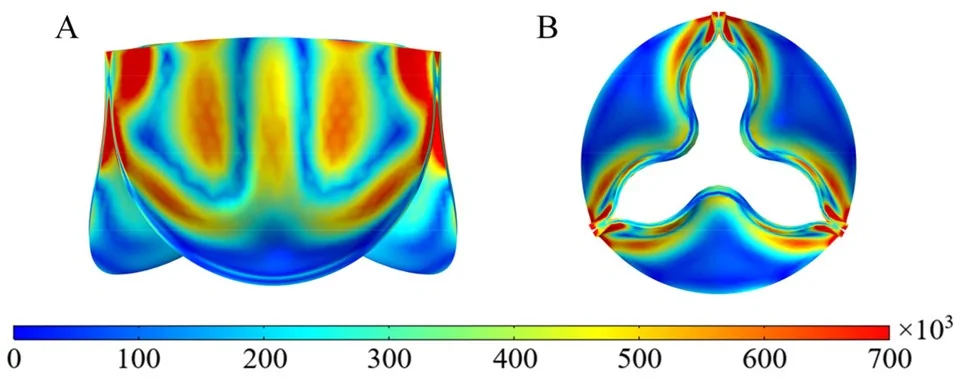
Von mises stress (Pa) distribution and deformed configuration of the leaflets for AR = 1.16 at *t *= 0.14 s: (**A**) front view, (**B**) top view.

**Figure 14:**
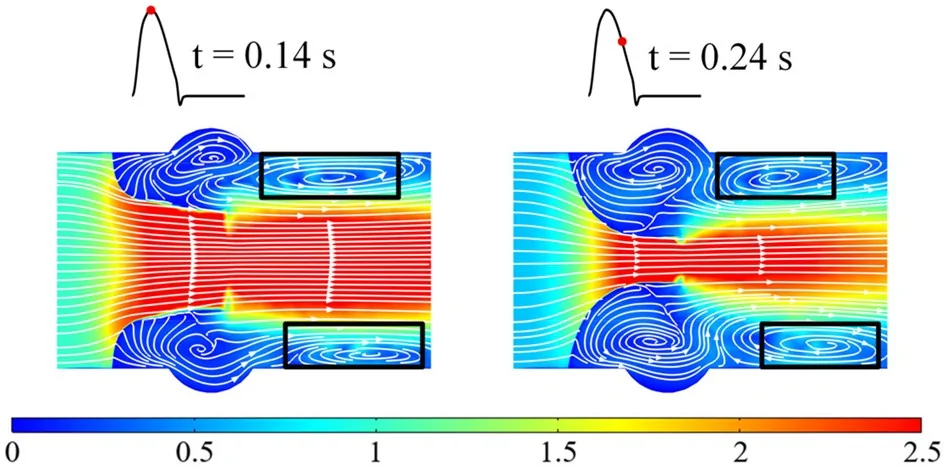
Velocity magnitude (m/s) distribution and streamlines on the XZ cross-section for the AR = 1.16 at *t *= 1.14 s and 0.24 s.

**Figure 15:**
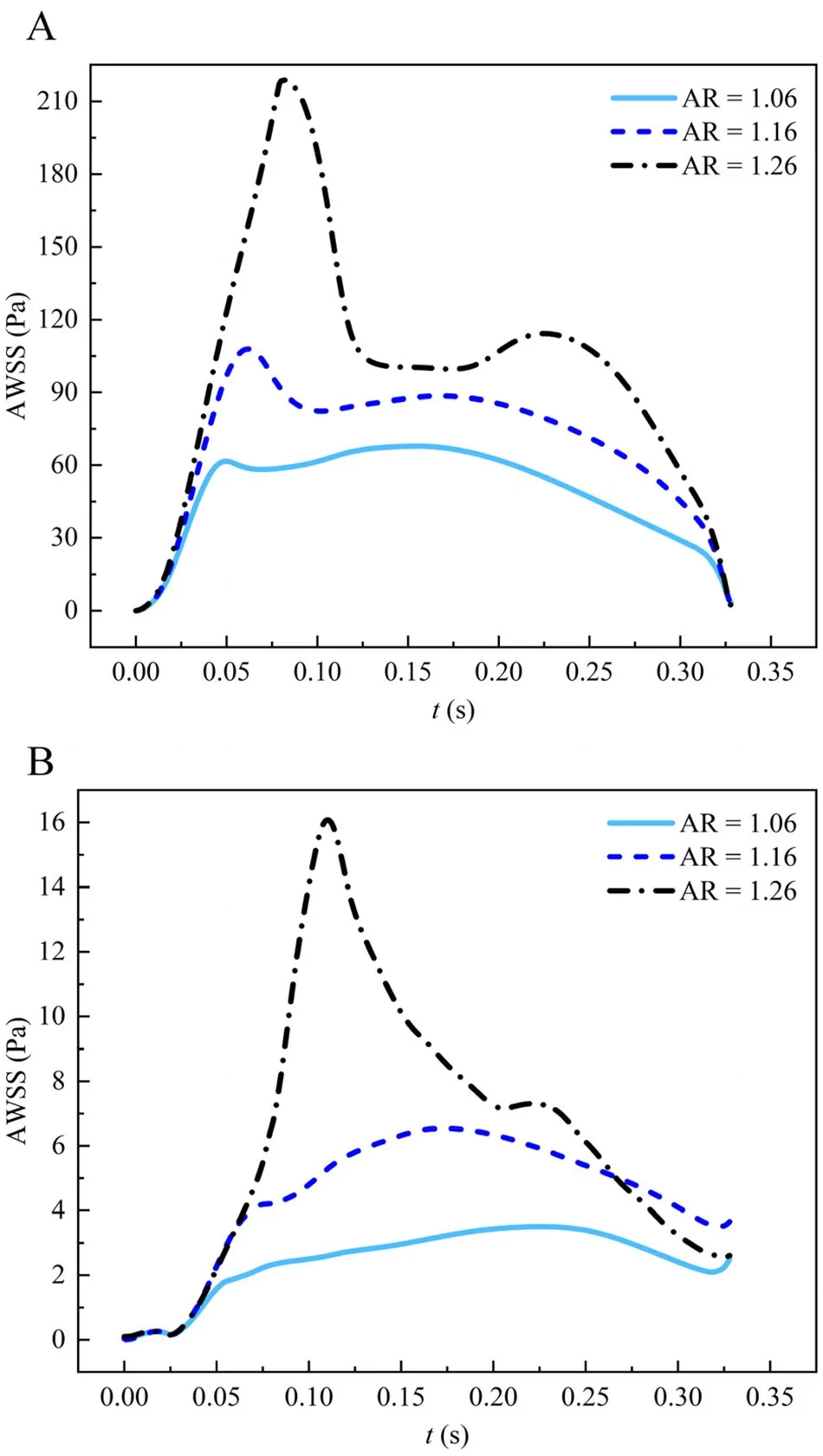
AWSS During systole on (**A**) the ventricular side and (**B**) the aortic side.

These changes are closely associated with the increase in leaflet AR. In the valve design, the entire lower edge of the leaflet is constrained. As the AR increases, the leaflet height increases, resulting in a larger constrained region under the fixed boundary condition and consequently limiting the effective deformation capability of the leaflet. This restriction reduces the GOA of the leaflets. According to the continuity principle, the reduced GOA leads to an increase in blood flow velocity, which subsequently elevates the TPG, thereby enhancing convective effects and amplifying the asymmetry of the flow field. Under the high AR condition (AR = 1.26), the interaction between leaflet deformation and flow dynamics further promotes asymmetric leaflet deformation and jet deviation. During valve closure, the increased constraint imposed by the fixed lower edge further limits leaflet deformation, resulting in incomplete or delayed coaptation. The resulting transient residual flow passages contribute to the increased RF.

### Non-Newtonian effects of blood flow

The shear-thinning behavior of blood cannot be captured by Newtonian fluid models [62]. Therefore, in this section, the Carreau–Yasuda model is employed to analyze the non-Newtonian effects based on the valve model with AR = 0.86. The viscosity curve corresponding to the parameter set adopted in the present study is shown in Figure 16. When blood is modeled as a Carreau–Yasuda non-Newtonian fluid, its viscosity decreases as the shear rate increases and approaches the viscosity of a Newtonian fluid. At low shear rates (*γ* < 100 s^−1^), the viscosity of non-Newtonian blood can reach 1.3–1.7 times that of a Newtonian fluid, resulting in slower blood flow and increased residence time.

**Figure 16:**
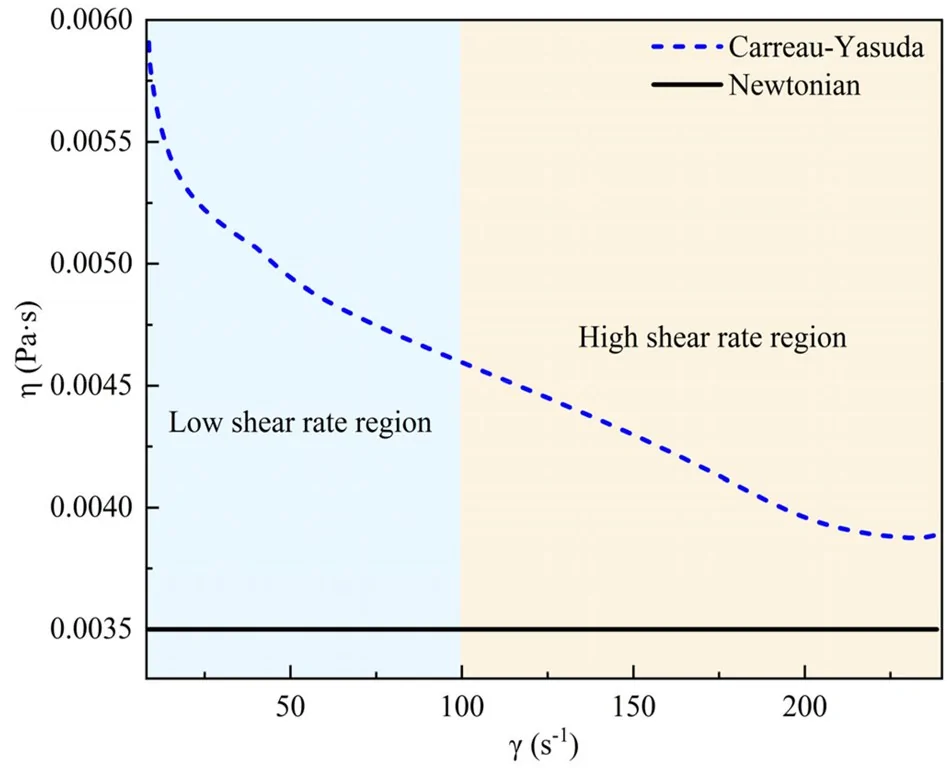
Viscosity of Newtonian and non-Newtonian fluids.

Tables 5 and 6 compare the maximum WSS (WSS_max_) on the valve and the AWSS on the ventricular surface for the two blood constitutive models at three times: early systole (0.04 s), the systolic peak (0.14 s) and mid-systole (0.24 s). The results indicate that the non-Newtonian effect has a significantly influences only on the local peak WSS. Specifically, the maximum WSS on the valve produced by the two fluid models during early systole differs by 5.15%, whereas the difference in the AWSS is only 0.11%. By *t *= 0.14 s, the differences are negligible, indicating that the non-Newtonian effect of blood diminishes at this point. After entering the mid-systole phase, the non-Newtonian effect gradually increases.

**Table 5:** Maximum WSS of the ventricular surface: Newtonian vs. Carreau–Yasuda model.

*t* (s)	WSS_max_ (Pa)-Newtonian	WSS_max_ (Pa)-Carreau–Yasuda	Error (%)
0.04	84.61	88.97	5.15
0.14	118.01	118.03	0.02
0.24	86.84	86.90	0.07

**Table 6:** AWSS of the ventricular surface: Newtonian vs. Carreau–Yasuda model.

*t* (s)	AWSS (Pa)-Newtonian	AWSS (Pa)-Carreau–Yasuda	Error (%)
0.04	28.11	28.14	0.11
0.14	40.10	40.14	0.07
0.24	25.38	25.39	0.03

This pattern of variation indicates that a simplified Newtonian model can be used to approximate the high-shear-rate phase of the cardiac cycle (e.g. peak systole); however, during the low-shear-rate period of early systole and the subsequent diastole, non-Newtonian effects of blood should be taken into account; otherwise, the local maximum WSS on the valve surface will be underestimated.

## Conclusion

In this study, the hemodynamic and mechanical behavior of the AV was investigated using a high-fidelity two-way FSI framework based on the ALE method. Within this framework, the valve is modeled as a hyperelastic, nearly incompressible shell structure. Physiologically appropriate flow conditions are imposed, including the inlet velocity profile derived from Doppler imaging data and the outlet pressure condition determined by the Windkessel model. In addition, a periodicity-convergent initial state is employed to account for the physiological pre-stress and initial flow conditions. The model is validated against experimental and published numerical results, and the results demonstrate good agreement. The effects of AR and blood non-Newtonian behavior are systematically analyzed, providing insights for prosthetic valve design and optimization. The main conclusions are as follows:

- An increasing leaflet AR leads to a greater blood flow velocity magnitude and a pronounced alteration in flow dynamics, with a higher AR promoting jet deflection and the development of asymmetric and more complex flow structures.
- Increased AR results in increased leaflet von Mises stress, WSS, TPG and RF, accompanied by a reduction in the GOA, indicating progressive stenotic behavior and an increased tendency toward regurgitation. Notably, the variation trends of the stress magnitude and GOA of the valve with increasing AR are opposite to the results of the pure structural mechanics analysis, highlighting the importance of FSI in valve performance evaluation.
- Non-Newtonian effects significantly affect the maximum WSS in low shear rate regions but have a limited influence on the AWSS. At the peak flow state, blood behavior approaches that of a Newtonian fluid.

We note that shell elements are computationally efficient and can capture the overall deformation behavior effectively but fall short in terms of contact treatment and anisotropic material representation. In addition, the present study does not account for turbulence effects, which may influence the detailed hemodynamic characteristics. Future work will focus on incorporating anisotropic features in hyperelastic shell models, improved contact algorithms and appropriate turbulence modeling.

## Supplementary Material

rbag167_Supplementary_Data
