# Supplementary material for: Fluid–structure interaction analysis of aortic valve dynamics: effects of aspect ratio
Source: Regen Biomater. 2026 Aug 5;13:rbag167. doi: 10.1093/rb/rbag167 (PMC13644530; doi:10.1093/rb/rbag167)
Supplement: Supplementary file 1 [file rbag167_supplementary_data.docx]

# Supplementary data A

To incorporate the physiological pre-stress that is typically absent in the initial zero-stress state of the leaflets, we perform FSI simulations for four cycles to determine a realistic prestress distribution.

Table A 1 summarizes the maximum leaflet von Mises stress (), displacement magnitude () and blood flow velocity magnitude () at the start of each cardiac cycle. The quantitative results reveal that the commonly adopted initial condition with zero-valve stress, deformation and velocity deviates notably from the physiological reality. Moreover, from the second cardiac cycle onward, only minor variations are observed in these quantities between consecutive cycles. The maximum relative differences in the von Mises stress and displacement values of the valve are less than 1.07% and 2.64%, respectively, whereas the peak blood flow velocity value remains constant at 0.063 m/s across all cycles. Furthermore, Figure A 1 presents the variation of the average wall shear stress (AWSS) and maximum wall shear stress (WSS) on the aortic side of the leaflets during the early acceleration phase (0.00–0.03 s) across four cardiac cycles. The Figure shows that the results in the first cycle deviate significantly from those in the subsequent three cycles, with maximum differences of 73.9434% for AWSS and 21.9914% for WSS. From the second cycle onward, the curves from different cycles almost completely overlap. Notably, at the initial instant, the values of AWSS and WSS are zero only in the first cycle, whereas non-zero initial values are observed in the subsequent cycles. Conclusions can be drawn from the above analysis that the periodicity-convergence initial condition is determined, i.e., the start of the second and the subsequent cardiac cycle. In the present analysis, computation results are recorded from the start of the second cardiac cycle to account for physiological pre-stress and initial flow conditions.

Table A 1. Numerical results under different numbers of cycles

| Cycle |  (Pa) | $\left\vert\mathbf{u}_{s}\right\vert_{\max}$ (mm) | $\left\vert\mathbf{v}_{f}\right\vert_{\max}$ (m/s) |
| --- | --- | --- | --- |
| First | 0.000 | 0.000 | 0.000 |
| Second | 194.069 | 4.590 × 10⁻⁷ | 0.063 |
| Third | 195.558 | 4.711 × 10⁻⁷ | 0.063 |
| Fourth | 196.143 | 4.674 × 10⁻⁷ | 0.063 |

Figure A 1. AWSS and maximum WSS on the aortic side of the leaflets over four cardiac cycles

# Supplementary data B

Mesh independence validation for the valve leaflet with an aspect ratio (AR) of 0.86 is conducted by three meshes with various resolutions, as detailed in Table B 1. The mesh definition for the valve (AR = 0.86) is shown in Figure B 1, where the mesh of the valve leaflet is globally refined, with additional local refinement applied along the entire lower edge region. Compared with the medium mesh, the boundary layer settings are modified. The coarse mesh uses a vascular wall boundary layer of 4 layers (stretching factor of 1.2; total thickness ≈ 0.11 mm) and a leaflet boundary layer of 2 layers (stretching factor of 1.15; total thickness ≈ 0.04 mm). In contrast, the fine mesh employs refined boundary layers with 16 layers on the vascular wall (stretching factor of 1.1; total thickness ≈ 0.72 mm) and 8 layers on the leaflet (stretching factor of 1.05; total thickness ≈ 0.19 mm).

The following validation metrics are selected for quantitative verification: the maximum von Mises stress, flow velocity magnitude, and geometric orifice area (GOA). The results from various mesh resolutions are compared to define the relative deviation.

$\delta=\frac{\left|X-X_{fine}\right|}{X_{fine}}\times100\%$ (B1)

Here, $X$ and $X_{fine}$ represent the results from the coarser and finest meshes, respectively.

Table B 1. Mesh resolution conditions for the leaflet (AR = 0.86) in the present verification

| Mesh resolution | number of fluid domain cells | number of solid domain cells |
| --- | --- | --- |
| coarse | 86,615 | 2,009 |
| medium | 138,666 | 2,637 |
| fine | 278,709 | 4,316 |

Figure B 1. Different mesh resolutions for the leaflet (AR = 0.86): (A) coarse, (B) medium, (C) fine

For the AR = 0.86 case, Table B 2 presents the maximum leaflet von Mises stress (), geometric orifice area ($\text{GOA}_{\max}$), and blood velocity ($\left|\mathbf{v}_{f}\right|_{\max}$). Table B 3 shows that the relative errors $\delta_{1}$ of the mechanical performance parameters between the coarse and fine meshes are substantial, with the relative errors of the maximum von Mises stress and blood flow velocity being 6.8772% and 6.9964%, respectively (both exceeding 5%). In contrast, the relative errors $\delta_{2}$ between the medium and fine meshes are all small, with the maximum relative error being only 1.1684%.

Table B 2. Numerical results at different mesh resolutions for the AR = 0.86 case of the leaflet

| Mesh resolution |  (Pa) | $\text{GOA}_{\max}$ (mm^2^) | $\left\vert\mathbf{v}_{f}\right\vert_{\max}$ (m/s) |
| --- | --- | --- | --- |
| coarse | 8.2086×10^5^ | 287.2869 | 2.3169 |
| medium | 7.6191×10^5^ | 291.7788 | 2.1401 |
| fine | 7.6804×10^5^ | 291.5588 | 2.1654 |

Table B 3. Relative errors of coarse and medium mesh results for the AR = 0.86 case with respect to the fine mesh

| Relative errors |  | $\text{GOA}_{\max}$ | $\left\vert\mathbf{v}_{f}\right\vert_{\max}$ |
| --- | --- | --- | --- |
| $\delta_{1}$ (%) | 6.8772 | 1.4652 | 6.9964 |
| $\delta_{2}$ (%) | 0.7981 | 0.0755 | 1.1684 |

For valve leaflets with aspect ratios (AR) of 1.06 and 1.26, the same meshing strategy as that used for the AR = 0.86 case is adopted, and corresponding fluid–structure interaction (FSI) simulations are performed. Table B 4 and Table B 5 present the numerical results for AR = 1.06 and AR = 1.26, respectively, while Table B 6 and Table B 7 summarize the relative errors among different mechanical parameters. These results verify that the medium mesh is capable of providing mesh-independent results; therefore, the medium mesh is uniformly adopted for all subsequent analyses in this paper.

Table B 4. Numerical results at different mesh resolutions for the AR = 1.06 case of the leaflet

| Mesh resolution |  (Pa) | $\text{GOA}_{\max}$ (mm^2^) | $\left\vert\mathbf{v}_{f}\right\vert_{\max}$ (m/s) |
| --- | --- | --- | --- |
| coarse | 9.8265×10^5^ | 191.0361 | 2.9853 |
| medium | 9.6246×10^5^ | 175.8483 | 3.0433 |
| fine | 9.6325×10^5^ | 172.9837 | 3.0937 |

Table B 5. Numerical results at different mesh resolutions for the AR = 1.26 case of the leaflet

| Mesh resolution |  (Pa) | $\text{GOA}_{\max}$ (mm^2^) | $\left\vert\mathbf{v}_{f}\right\vert_{\max}$ (m/s) |
| --- | --- | --- | --- |
| coarse | 1.4831×10^6^ | 86.8933 | 4.9156 |
| medium | 1.3227×10^6^ | 83.2825 | 5.2299 |
| fine | 1.3350×10^6^ | 82.4464 | 5.3053 |

Table B 6. Relative errors of coarse and medium mesh results for the AR = 1.06 case with respect to the fine mesh

| Relative errors |  | $\text{GOA}_{\max}$ | $\left\vert\mathbf{v}_{f}\right\vert_{\max}$ |
| --- | --- | --- | --- |
| $\delta_{1}$ (%) | 2.0140 | 10.4359 | 3.5039 |
| $\delta_{2}$ (%) | 0.0820 | 1.4844 | 1.6291 |

Table B 7. Relative errors of coarse and medium mesh results for the AR = 1.26 case with respect to the fine mesh

| Relative errors |  | $\text{GOA}_{\max}$ | $\left\vert\mathbf{v}_{f}\right\vert_{\max}$ |
| --- | --- | --- | --- |
| $\delta_{1}$ (%) | 11.0936 | 5.3937 | 7.3455 |
| $\delta_{2}$ (%) | 0.9213 | 1.0141 | 1.4212 |

# Supplementary data C

The time step sensitivity is evaluated using time steps of 0.001 s and 0.005 s. Table C 1 presents the results for key parameters; the relative errors for the maximum von Mises stress () of the valve, the maximum geometric orifice area ($\text{GOA}_{\max}$), and the maximum blood velocity magnitude ($\left|\mathbf{v}_{f}\right|_{\max}$) are 0.9502%, 0.9987%, and 0.4486%, respectively. Figure C 1A and Figure C 1B compare the blood flow field distributions at *t* = 0.24 s for time steps of 0.001 s and 0.005 s, respectively. The area inside the red box indicates that the local blood velocity through the valve is higher when the time step is 0.005 s. As indicated by the black arrow, the downstream near-wall region displays regular, nearly parallel laminar flow at a time step of 0.005 s, but deviates slightly and tends to form vortices at a time step of 0.001 s. Although the coarser time step (0.005 s) shows deviations of less than 1% in key parameters compared with the finer time step (0.001 s), it fails to adequately capture transient flow dynamics. In contrast, a time step of 0.001 s accurately resolves the dynamic process. Accordingly, all the simulations in this paper employ a time step of 0.001 s.

Table C 1. Numerical results at different time steps

| Time step (s) |  (Pa) | $\text{GOA}_{\max}$ (mm^2^) | $\left\vert\mathbf{v}_{f}\right\vert_{\max}$ (m/s) |
| --- | --- | --- | --- |
| 0.001 | 7.6191×10^5^ | 291.7788 | 2.1401 |
| 0.005 | 7.6915×10^5^ | 288.8649 | 2.1497 |

Figure C 1. Velocity magnitude (m/s) distribution and streamlines on the XZ cross-section at different time steps at *t* = 0.24 s: (A) time step = 0.001 s, (B) time step = 0.005 s

# Supplementary data D

To verify the reliability of the proposed numerical model and computational framework, the leaflet geometry and simulation conditions reported by Liu et al. [1] are reproduced for validation. The leaflet radius is set to 9 mm, while the aortic root is modeled without the sinus structure. The leaflets are described using a three-parameter Mooney–Rivlin hyperelastic constitutive model with material parameters of *c*_10_ = 0.17 MPa, *c*_01_ = 2.17 MPa, and *c*_11_ = 3.43 MPa. A Poisson's ratio of 0.48 is specified to approximate the nearly incompressible mechanical behavior of the leaflet tissue.

In the boundary conditions, a time-dependent total pressure profile consistent with that reported by Liu et al. [1] is prescribed at the inlet (see Figure D 1), while a constant zero-gauge pressure is imposed at the outlet to ensure consistent driving conditions for the flow. The numerical simulations are performed using the arbitrary Lagrangian–Eulerian (ALE) method. The valve leaflets are modeled as hyperelastic, nearly incompressible thin shells to accurately capture their large deformation behavior, and a fully coupled fluid–structure interaction (FSI) framework is employed to resolve the interaction between the fluid and structural domains.

Figure D 1. Transient boundary condition for blood flow: inlet total pressure

Figure D 2 presents the deformed configuration of the leaflets at full opening and the corresponding von Mises stress distribution. The overall deformation pattern of the leaflets shows good agreement with the results reported by Liu et al. [1]. Under the fully opened state, stresses are primarily concentrated in the belly region of the leaflet as well as along the entire lower edge. In the present FSI simulation for the supplementary validation case, the maximum von Mises stress of the leaflet is 492 kPa, compared with approximately 500 kPa reported in the literature, yielding a relative error of 1.6%. This indicates a good agreement in the predicted stress levels. Figure D 3 further compares the wall shear stress (WSS) distributions on the ventricular and aortic sides of the valve leaflets. The results show that the spatial distribution patterns are generally consistent with those reported by Liu et al. [1] for a leaflet thickness of 0.2 mm. On the ventricular side, WSS is predominantly concentrated in the belly region of the leaflet, with a relatively higher overall magnitude. In contrast, on the aortic side, WSS is mainly localized near the leaflet tip region. In the present study, the maximum WSS values on the ventricular and aortic sides are 10.6 Pa and 1.4 Pa, respectively, which are in good agreement with those reported in the reference study. This comparison indicates that the present FSI framework is capable of accurately reproducing the key near-wall hemodynamic characteristics of the valve leaflets.

Figure D 2. Von Mises stress (Pa) distribution and deformed configuration of the valve: (A) top view of the fully opened state, (B) von Mises stress distribution of a single leaflet

Figure D 3. WSS (Pa) distribution: (A) the ventricular side, (B) the aortic side

Over a complete cardiac cycle, the blood flow velocity exhibits a typical unsteady behavior. The flow gradually accelerates from the initial stage, develops into a jet-like structure (see Figure D 4), and then progressively decays as the pressure gradient decreases. The peak velocity occurs at approximately 0.3 s, with a maximum value of about 1.5 m/s, which is in good agreement with the reported values in the literature. This further validates the reliability of the present model in predicting the overall hemodynamic response of the system.

Figure D 4. Velocity magnitude (m/s) distribution at *t* = 0.3 s

# Reference

1.	Liu X, Lee A, Wang Y, Hoang TP, Yee KS, Mosse L, Karajan N, Winlaw DS, Naficy S, Fletcher DF. Fluid-structure interaction analysis of bioinspired polymeric heart valves with experimental validation. *Comput Methods Programs Biomed* 2025;**268**:108839.
